# Distinct Neural Substrates for Maintaining Locations and Spatial Relations in Working Memory

**DOI:** 10.3389/fnhum.2016.00594

**Published:** 2016-11-24

**Authors:** Kara J. Blacker, Susan M. Courtney

**Affiliations:** ^1^Department of Psychological and Brain Sciences, Johns Hopkins University, BaltimoreMD, USA; ^2^Department of Neuroscience, Johns Hopkins University School of Medicine, BaltimoreMD, USA; ^3^F.M. Kirby Research Center for Functional Brain Imaging, Kennedy Krieger Institute, BaltimoreMD, USA

**Keywords:** working memory, spatial, relational processing, load effects, fMRI

## Abstract

Previous work has demonstrated a distinction between maintenance of two types of spatial information in working memory (WM): spatial locations and spatial relations. While a body of work has investigated the neural mechanisms of sensory-based information like spatial locations, little is known about how spatial relations are maintained in WM. In two experiments, we used fMRI to investigate the involvement of early visual cortex in the maintenance of spatial relations in WM. In both experiments, we found less quadrant-specific BOLD activity in visual cortex when a single spatial relation, compared to a single spatial location, was held in WM. Also across both experiments, we found a consistent set of brain regions that were differentially activated during maintenance of locations vs. relations. Maintaining a location, compared to a relation, was associated with greater activity in typical spatial WM regions like posterior parietal cortex and prefrontal regions. Whereas maintaining a relation, compared to a location, was associated with greater activity in the parahippocampal gyrus and precuneus/retrosplenial cortex. Further, in Experiment 2 we manipulated WM load and included trials where participants had to maintain three spatial locations or relations. Under this high load condition, the regions sensitive to locations vs. relations were somewhat different than under low load. We also identified regions that were sensitive to load specifically for location or relation maintenance, as well as overlapping regions sensitive to load more generally. These results suggest that the neural substrates underlying WM maintenance of spatial locations and relations are distinct from one another and that the neural representations of these distinct types of spatial information change with load.

## Introduction

Working memory (WM) is the ability to actively maintain and manipulate relevant information that is not currently available as sensory input. WM is critical for learning new skills, solving novel tasks, and guiding goal-directed behavior. WM has traditionally been associated with maintenance of stimulus-specific features, such as phonological, spatial or object-based information (Baddeley and Hitch, 1974; Baddeley and Logie, 1999). For example, a body of work has described the role of prefrontal cortex (PFC; for a review see, ; Levy and Goldman-Rakic, 2000; Curtis and D’Esposito, 2003; D’Esposito, 2007), posterior parietal cortex (PPC; e.g., Todd and Marois, 2004), and primary visual cortex (e.g., Serences et al., 2009) in the maintenance of visuospatial information in WM. While much research has focused on whether the neural mechanisms underlying WM maintenance of spatial vs. object-based vs. verbal information are distinct or overlapping (e.g., Shah and Miyake, 1996; Duncan and Owen, 2000; Levy and Goldman-Rakic, 2000; Miller and Cohen, 2001; Sala and Courtney, 2007; Chein et al., 2011; Yeo et al., 2014), one commonality exists for all of these modalities: each involves the maintenance of concrete, sensory-based information in WM. However, many tasks require that other types of information that are more “abstract” be maintained, such as relationships, rules, or strategies. These forms of abstract information must be extracted from sensory stimuli in some way and then maintained in WM. Recent work has begun to suggest that maintaining abstract, non-sensory information in WM relies on distinct neural mechanisms than maintaining concrete, sensory information in WM (Badre, 2008; Montojo and Courtney, 2008; Ackerman and Courtney, 2012; Bahlmann et al., 2014; Ikkai et al., 2014; Libby et al., 2014; Blacker et al., 2016).

This distinction between abstract and sensory information can be made in many different forms. For example, here we focus on spatial information and contrast WM for locations vs. spatial relations. However, previous work has also made this distinction by comparing number vs. mathematical operations (Montojo and Courtney, 2008) and visual features vs. semantic properties of objects (Lee et al., 2013). In the current study, we are investigating differences in WM maintenance for spatial locations and spatial relations. A spatial location is an absolute coordinate of an object in space and thus considered concrete or sensory. Whereas a spatial relation is the position of an object relative to another object (e.g., above/below) and thus is not tied specifically to the sensory location and is therefore more “abstract.” Another possible conception of locations and relations from the broader literature may be in terms of egocentric vs. allocentric reference frames. For example, while a location can be defined in egocentric coordinates (i.e., relative to the observer), a relation may be considered allocentric (i.e., relative to another object). Indeed, there is evidence for distinct neural mechanisms supporting egocentric and allocentric strategies in spatial navigation in humans (Jordan et al., 2004) and spatial perception in primates (e.g., Andersen et al., 1985) and rodents (for a review see, Moser et al., 2008). These results support the idea that the distinction between locations and relations is not merely a transformation to a different coordinate system, but two distinct types of spatial information, supported by different neural substrates.

Recent functional magnetic resonance imaging (fMRI) evidence suggests that sub-regions of PPC and PFC are differentially active during maintenance of abstract vs. item-specific information (Montojo and Courtney, 2008; Ackerman and Courtney, 2012). While there is evidence from fMRI studies that there may be some degree of domain specificity within the abstract WM systems, such as for spatial vs. non-spatial relationships, or for mathematical operations vs. magnitude comparisons, there remains a broader distinction in all of these cases for concrete, sensory vs. abstract, non-sensory information that is consistent across studies. For example, Ackerman and Courtney (2012) demonstrated that WM for abstract spatial relations resulted in more activity in anterior portions of PFC and PPC, whereas WM for spatial locations resulted in more activity in posterior portions of those regions. This double dissociation suggests that the neural mechanisms by which sensory and non-sensory information are maintained in WM may be distinct, or at least that they are handled as distinct types of representations. These previous studies, however, did not examine the differential role that early visual cortex may play in maintaining retinotopic information when spatial locations vs. spatial relations are maintained in WM. One recent study suggests that spatial coding in early visual cortex is not obligatory or automatic and that the strategy one applies to a visual memory item may change the neural code (Vicente-Grabovetsky et al., 2014). These results support our hypothesis here that the neural codes maintained for sensory vs. non-sensory spatial information are distinct.

Previous studies examining how visuospatial information is maintained in WM have shown that in addition to sustained PPC and PFC activity, WM maintenance yields sustained activation of early visual cortex. Specifically, early visual areas seem to retain specific information about the stimulus features held in WM, over delay periods when no physical stimulus is present (Ester et al., 2009, 2013; Harrison and Tong, 2009; Serences et al., 2009; Riggall and Postle, 2012; Emrich et al., 2013). However, it is unclear what role visual cortex plays when a specific sensory stimulus cannot be anticipated at test and a relationship must instead be maintained in WM. If a task requires that the spatial relationship between two objects be maintained in WM, and the absolute spatial coordinates of those objects become task-irrelevant, then the fate of those original sensory codes is unknown. One recent study suggests that when a visual object is encoded into WM and the relevant feature of that object is unknown (e.g., semantic, visual, and verbal) then multiple mental codes are maintained; however, when the relevant feature is known (e.g., semantic), the other features (e.g., visual and verbal) are discarded from memory (Lewis-Peacock et al., 2014). While this study examined this question of mental codes with a variety of sensory features, it remains unclear if the same rule applies to sensory and non-sensory memoranda that are both spatial in nature. It is possible that relational information can only be represented as a hierarchical derivation built upon sensory-specific representations in WM. In that case, the sensory information (e.g., retinotopic information) about the sample stimulus would continue to be maintained during maintenance of relational information, even though the sensory information is not directly relevant for performing the task and may even interfere with optimal performance. Alternatively, sensory and abstract representations may be independent of one another rather than hierarchical, which would follow from the study by Lewis-Peacock et al. (2014).

Two recent electroencephalography (EEG) studies began to investigate this question and found that maintaining abstract spatial relations in WM resulted in increased alpha (8–13 Hz) power over posterior brain regions, as compared to maintaining concrete, spatial locations in WM (Ikkai et al., 2014; Blacker et al., 2016). Recent evidence suggests that alpha oscillations play a direct role in selective attention and WM, particularly in the suppression of brain regions responsible for processing task-irrelevant information (Worden et al., 2000; Fu et al., 2001; Kelly et al., 2006; Jokisch and Jensen, 2007; Rihs et al., 2007; Sauseng et al., 2009; Jensen and Mazaheri, 2010; van Dijk et al., 2010; Bengson et al., 2012). Therefore, Ikkai et al. (2014) concluded that more posterior alpha power during maintenance of spatial relations was indicative of suppression of sensory cortex. However, given the low spatial resolution of EEG, it is difficult to specifically localize this increase in alpha oscillations to visual cortex or to determine whether any sensory information was preserved when spatial relations were maintained in WM.

Here we present two fMRI experiments that manipulate whether participants were required to maintain spatial locations or spatial relations in WM. Importantly, we set up the task so that the location or relation information was encoded from identical visual input. Previous work has shown that the neural correlates of WM can be dissociated based on behavioral goals (Lee et al., 2013). Here we predicted that spatial relations would be maintained in WM via distinct neural mechanisms compared to spatial locations. Specifically, we expected less quadrant-specific activation in early visual cortex when a spatial relation was maintained compared to a spatial location. Based on previous work, we anticipated differential neural substrates beyond visual cortex to also be activated when spatial locations vs. spatial relations were maintained in WM. Specifically, we expected that maintaining concrete spatial locations would be associated with greater activity in PPC and posterior dorsolateral PFC regions, which have been previously shown to be involved in spatial WM (Courtney et al., 1996, 1998; Owen et al., 1996; Ungerleider et al., 1998; Levy and Goldman-Rakic, 2000; Curtis et al., 2004). We expected that WM for spatial relations would differentially activate other regions, such as anterior PFC (e.g., Ackerman and Courtney, 2012) or the medial temporal lobes (MTL; e.g., Libby et al., 2014). Finally, in Experiment 2, we manipulated the number of to-be-remembered spatial locations and relations in order to identify whether regions active for maintaining each of these two types of information were also sensitive to load for relations or locations, which serves as an indication of representation of information within an information-type.

## Materials and Methods

### Participants

In Experiment 1, 32 adults (8 male) participated and in Experiment 2, 33 different adults (10 male) participated. All participants (18–30 years of age) participated for monetary compensation. All participants had normal or corrected-to-normal vision and were right-handed, non-smokers, in good health with no reported history of head injury, neurological or mental disorders, or drug/alcohol abuse, and no current use of medications that target central nervous system or cardiovascular function. All participants gave written informed consent approved by the Institutional Review Boards of Johns Hopkins University and the Johns Hopkins Medical Institutions.

### Behavioral Methods

#### Experiment 1 WM Task and Procedure

**FIGURE 1:**
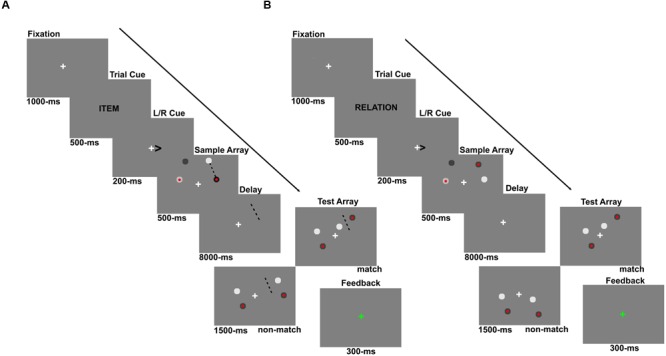
**Experiment 1 trial schematics showing example (A)** Location and **(B)** Relation trials. Participants were always cued to make a covert shift of attention to either the left or right hemifield via an arrow cue. For Location trials, participants were cued “Item” which instructed them to imagine a line between the two sample array circles (shown here only for illustration purposes) and hold the location of that line in memory over the delay. At test, participants indicated whether the two test circles straddled the imaginary line. For Relation trials, participants were cued “Relation” which instructed them to remember the relative vertical relationship of the two circles, using the red-center as the reference. At test, participants indicated whether the test circles had the same relationship or not.

As shown in **Figure 1**, all stimuli were presented on a 50% gray background. A trial began with a 1000 ms fixation cross (0.12°), presented in the middle of the screen. Next, a 500 ms cue indicated whether participants were to remember a particular location (“Location trial,” cued by the word “Item”) or the relative spatial relationship between the items presented (“Relation trial,” cued by the word “Relation”). This task cue was followed by a 200 ms arrow cue, which indicated whether the left or right hemifield should be attended. A sample array was then presented for 500 ms, which contained four circles of varying shades of gray (each subtending 0.3° of visual angle), 2 in each hemifield. One circle in each hemifield contained a red center (0.1°). After a 8 s delay period, four more circles, two in each hemifield, were presented as the test array for 1500 ms during which the participant entered a response. The test array was followed by a 200 ms feedback period where the fixation cross turned green for a correct response, red for an incorrect response, and blue if the response was slower than 1500 ms. After every 10 trials, the word “REST” was presented in the center of the screen for 12 s.

For Location trials (**Figure 1A**), participants were instructed to draw an imaginary line segment from one circle to the other and maintain the location of that line in memory over the delay period. These instructions were used to encourage participants to encode the exact spatial coordinates of one concrete object (i.e., the imaginary line segment). At test, participants were asked to decide whether or not the test circles were “straddling” the imaginary line formed by the initial two circles. In other words, if a line segment was drawn between the two test circles, would that line intersect the initial line segment connecting the sample array circles. For Relation trials (**Figure 1B**), participants were instructed to encode and maintain the relative vertical positions of the two circles in the cued hemifield, using the one with the red center as a reference (i.e., is the other circle above or below the circle with the red center?). Upon test, participants indicated whether or not the circles in the test array had the same relative positions as the sample circles. For both trial types, participants pressed one button for a “match” response and another for a “non-match” response and these response key mappings were counterbalanced between participants.

There are a few crucial aspects of the task design worth elaborating on. First, regardless of trial type, participants were asked to encode and maintain one piece of information: either one spatial location (Location trials) or one spatial relationship (Relation trials), and they needed to manipulate the sensory information in some way to obtain that single piece of information in both tasks. Second, trial type was pseudo-randomly presented so participants could not predict what trial type they would see until the cue. Third, the sample array circles were always presented in either the top or bottom quadrant of the relevant hemifield. Each quadrant of the visual display spanned 4.75° × 3.78° visual angle and the edges of each quadrant were set 0.4° of visual angle off of the horizontal and vertical meridians. These quadrant dimensions were the same as those used for the flickering checkerboard in the quadrant localizer runs to identify voxels within retinotopically organized visual areas that responded to the spatial positions occupied by the stimuli in the WM runs. The test array circles were equally presented in either the same quadrant as the sample circles, the opposite quadrant as the sample circles, or split across the two quadrants. However, when the test circles were presented in the opposite quadrant as the sample circles, it would be impossible to have a match Location trial because the test circles could not straddle the line segment the participants imagined. Therefore, when the test circles appeared in the opposite quadrant as the sample circles for both Relation and Location trials, the correct response was always a non-match. This manipulation was used to ensure that while the information was encoded from only one quadrant, the participant could expect to be tested on information presented anywhere in the relevant hemifield for both types of trials. Finally, the task was designed to ensure that participants could not use one strategy (i.e., locations or relations) for both trial types. For example, if participants based their response to all Location trials on the relational information, accuracy would be at chance. In other words, 50% of trials would require the opposite response for the alternate trial type.

Participants completed a practice session on a separate day, prior to the fMRI session, in order to become familiar with the task. During the practice session, participants completed 200 trials (50% each trial type). During the fMRI session, participants completed four runs of 40 trials, for a total of 160 trials. Each run was 528 s in length.

#### Experiment 2 Working Memory Task and Procedures

**FIGURE 2:**
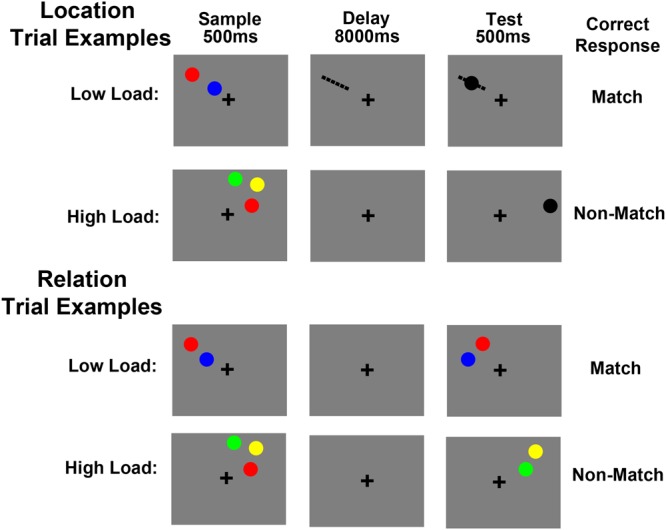
**Experiment 2 trial schematics showing example Location and Relation trials.** For low load Location trials participants were instructed to imagine a line between two sample circles (shown here only for illustration purposes), hold the location of that line in memory across a delay and then decide if a test circle fell in that location or not. For high load Location trials participants maintained the locations of three circles in memory and then decided if a test circle fell in one of those locations or in a completely new location. For low load Relation trials participants maintained the vertical relationship (above/below) of two sample circles and then decided if two test circles were in the same relationship. For high load Relation trials participants maintained the three vertical relationships between three sample circles and then decided if the circles of one of those pairs were presented in the same relationship at test.

Trial schematics for Experiment 2 are shown in **Figure 2**. A trial began with a 400 ms fixation cross (0.12°), presented in the middle of the screen. Next, a 500 ms cue indicated whether participants were to remember a particular location (“Location trial,” cued by the word “Item”) or the relative spatial relationship between the items presented (“Relation trial,” cued by the word “Relation”). A sample array was then presented for 500 ms, which contained two or three colored circles (each subtending 0.5° of visual angle). The presentation of the sample circles was restricted to one quadrant of the display. The color of each circle was chosen randomly without replacement from red, green, yellow, and blue. After a 8 s delay period, a test array was displayed for 500 ms. Participants had an additional 1000 ms after the offset of the test array to respond, during which there was only a fixation cross on the screen. Finally, a 100 ms feedback display was presented. After every 13 trials, the word “REST” was presented in the center of the screen for 11 s.

For both trial types, there was a low load (i.e., sample array contained two colored circles) and a high load (i.e., sample array contained three colored circles) condition. For Location trials (**Figure 2**), under low load, participants were instructed to imagine a line segment from one circle to the other and maintain the location of that line in memory. At test, participants were asked to decide whether or not the black test circle was on the imaginary line. For Location trials, under high load, participants were instructed to remember the absolute locations of the three sample circles. At test, participants were asked to decide whether or not the black test circle was in one of the three sample locations or in a completely new location. Of note, the low load Location trials required that only the location of the imaginary line be maintained in WM and compared at test, which allows for direct comparison of the low and high load Location trials (i.e., both involve maintaining a location or locations). Whereas the imaginary line instruction may appear to encourage participants to maintain the location, orientation, and the length of the line segment, the experimenter explicitly instructed the participant to only remember the location. Also, the test array stimuli during non-match trials were presented sufficiently far from the location of the imaginary line that even if the participant misremembered the orientation or length, they would still get the correct answer if they remembered the location, making it the only relevant feature. Thus, despite the instructional differences between our low and high load trials for Location, the functional task demands are identical with the exception of the number of to-be-remembered locations.

For Relation trials (**Figure 2**), under low load, participants maintained the relative vertical positions of the two circles in the cued hemifield (e.g., red is above blue). Upon test, participants indicated whether or not the circles in the test array had the same relative positions as the sample circles. For Relation trials, under high load, participants were instructed to encode and maintain the three possible vertical relationships between the sample circles (e.g., green is above yellow, yellow is above red, red is below green). As with low load, at test, participants indicated whether or not the circles in the test array had the same relative positions as the sample circles. Note, which of the three relationships was tested was unpredictable, which forced participants to maintain all three relationships during the delay period. For all trial types, participants pressed one button for a “match” response and another for a “non-match” response and these response key mappings were counterbalanced across participants.

Similar to Experiment 1, regardless of trial type, under low load, participants were asked to encode and maintain one piece of information: either one spatial location (Location trials) or one spatial relationship (Relation trials) and under high load, participants were asked to encode and maintain three pieces of information: either three spatial locations or three spatial relations. Trial type was pseudorandomly presented. Load was uncued, so the participants were unaware of the load until the sample array appeared. Third, the sample array circles were always presented in one quadrant of the display, where each quadrant spanned 4.0° × 3.8° visual angle and the edges were set 0.2° of visual angle off of the horizontal and vertical meridians^1^. These quadrant dimensions were the same as those used for the flickering checkerboard in the quadrant localizer runs to identify voxels within retinotopically organized visual areas that responded to the spatial positions occupied by the stimuli in the WM runs. The test array circles were always presented in the same quadrant as the sample circles. As in Experiment 1, the task was designed to ensure that participants could not use one strategy (i.e., locations or relations) for both trial types.

Participants completed a practice session on a separate day that consisted of 64 trials (16 of each trial type/load combination). During the fMRI session, participants completed 6 runs of 48 trials, for a total of 288 trials. Each run was 561 s in length.

### General fMRI Methods

#### fMRI Data Acquisition and Analysis

MRI scanning was carried out on a Phillips Achieva 3T scanner in the F.M. Kirby Research Center for Functional Brain Imaging at the Kennedy Krieger Institute (Baltimore, MD, USA). Blood oxygenation level-dependent (BOLD) changes in the MRI signal were collected using a 32-channel SENSE head coil (MRI Devices, Inc., Waukesha, WI, USA). Stimuli were presented on a laptop running MATLAB (The MathWorks; Natick, MA, USA) and Psychtoolbox (Brainard, 1997; Pelli, 1997) software. A liquid crystal display projector located outside of the scanning room back-projected the stimuli onto a screen located inside of the bore of the scanner. Participants viewed the stimuli via a mirror mounted to the top of the head coil. Responses were made with left- or right- thumb presses on hand-held button boxes connected via fiber-optic cables to a Cedrus RB610 response pad (Cedrus, San Pedro, CA, USA).

Anatomical images were acquired using a magnetization prepared rapid gradient echo (MPRAGE) *T*_1_-weighted sequence that yielded images with a 1mm resolution (150 coronal slices, TR = 7.9 ms, TE = 3.65 ms, flip angle = 8°). The functional *T*_2_*-weighted MR scans were sequential gradient echo, echo planar images (EPI; 35 axial slices, TR = 2000 ms, TE = 30 ms, flip angle = 70°, image matrix = 80 × 80, field of view = 240 mm, slice thickness = 2.5 mm with a 0.5 mm gap).

Using each participant’s MPRAGE scan, individual segmented and inflated cortical surface models were created in Freesurfer (Dale et al., 1999; Fischl et al., 1999) and then fMRI data were projected onto those surface models for analysis and visualization. Data were analyzed using the AFNI software package (Cox, 1996) and SUMA (Saad and Reynolds, 2012). Functional EPI data were phase shifted to correct for slice acquisition time and aligned to the second image of the run to correct for motion. Functional images were co-registered to anatomical images and then the aligned functional images were mapped onto each individual’s standardized cortical surface model per hemisphere. Individual participant data from the WM task runs only were spatially smoothed on the surface, using SUMA’s SurfSmooth command (Saad and Reynolds, 2012), to a resulting 4 mm full width of half maximum (FWHM). Data from the functional quadrant localizer runs (detailed below) were not smoothed. Finally, data were normalized as percent change from the run mean.

Our WM task in both experiments utilized an event-related design. Multiple regression analysis was performed on the time-series data at each surface node, for all nodes on the brain. There were separate event-related regressors for “sample,” “delay,” and “test” periods. For each of these trial event periods, there were separate regressors for Location and Relation trials, as well as for the quadrant of visual space in which the sample array appeared (i.e., Upper Left, Upper Right, Lower Left, and Lower Right), for a total of 8 different trial types. Experiment 2 contained additional regressors for each load (i.e., low and high), totaling 16 different trial types. In both experiments, these trial types were pseudorandomly ordered to ensure that each trial type was equally likely to follow any of the other trial types. Nuisance regressors included one regressor for incorrect trials and six regressors that modeled the motion parameters. Regressors were convolved with a γ function model of the BOLD response.

#### Independent Functional Localizer Scans

Participants completed two 320 s runs of a quadrant localizer task to independently identify voxels within retinotopically organized visual areas that responded to the spatial positions occupied by the stimuli in the WM runs. A contrast reversing checkerboard rectangle (flickering at 8 Hz) was presented covering the same bounds as the WM stimuli in each experiment (see below for details). The flickering rectangle was presented for 10 s in each quadrant in sequential counterclockwise order. Participants were asked to respond via a button press to a change in color (black to gray) of the fixation stimulus (i.e., a square; 0.2°) in order to ensure maintenance of central fixation throughout a run.

#### Region of Interest (ROI) Analyses

Using the quadrant localizer data, we delineated ventral and dorsal visual areas in each hemisphere, by testing an individual-level GLM on upper vs. lower quadrant presentations. We limited our ROIs to V1, V2, and V3 as determined by separate meridian mapping scans in Experiment 1 and using a probabilistic atlas of retinotopic visual areas (Wang et al., 2014) in Experiment 2. In both experiments, we analyzed data from the contralateral hemisphere from the presented sample array. Further, we were interested in the BOLD activity in the contralateral “corresponding” quadrant (i.e., dorsal regions for an lower hemifield array and ventral regions for an upper hemifield array) relative to the contralateral “opposite” quadrant (i.e., dorsal regions for an upper hemifield array and ventral regions for a lower hemifield array). More BOLD activity in the corresponding quadrant compared to the opposite quadrant would be indicative of a more retinotopically localized response. It is worth noting that previous studies have failed to find significantly above baseline BOLD activity during the delay period of visuospatial WM tasks (Tong and Nakayama, 1999; Serences et al., 2009), which is why here we focused on the relative activity of the corresponding and opposite quadrants in visual cortex. We considered V1, V2, and V3 together here, as we had no *a priori* expectation of these three areas showing differential responses during our WM delay periods in both experiments.

We averaged the event-related BOLD responses for each event type (Relation vs. Location, presented quadrant of visual space, and WM load in Experiment 2) for each ROI in each participant. As described above, we included separate regressors for the cue/sample, delay, and test periods. Because the delay always followed the sample, we made no attempt to distinguish between encoding and maintenance. While we would expect differences in encoding due to the cue for the different trial types, the sample stimuli themselves were identical for Location and Relation trials. Any differences in maintenance between Location and Relation trials were expected to be strongest during the delay period. Therefore, the analyses focused on differences in the delay period BOLD activity.

#### Whole-Brain Analysis

We also examined a whole brain analysis to isolate regions that were more sensitive to maintaining spatial relations vs. maintaining spatial locations in WM. Focusing on the delay period, we contrasted Relation trials vs. Location trials. We also used whole brain analysis in Experiment 2 to examine the effects of load within each trial type. Tests of node-wise significance were held to an uncorrected *p* < 0.01 and corrected for multiple comparisons via spatial extent of activation. Holding each cluster of nodes to an experiment-wise *p* < 0.05 required a minimum cluster size of 92 mm^2^, based on a Monte Carlo simulation with 1,000 iterations run via the SUMA software package, using the imposed smoothness of 4 mm FWHM. This smoothness value was greater than the measured smoothness of the residuals in the individual participant surface maps. Thus, our estimate of the minimum cluster size required to reach the statistical threshold is more conservative than it would have been using this alternative method of using the smoothness of residuals (Worsley et al., 1996; Kiebel et al., 1999). Of note, recent concerns about cluster correction have been raised (Eklund et al., 2016) that include AFNI’s 3dttest++ program used here. There are three pieces of evidence here that suggest our false positive rate is not inflated: (1) we used a smaller imposed smoothing (4 mm) than that used by Eklund et al. (2016), which makes our cluster threshold more conservative, (2) AFNI has made changes to address these problems (Cox et al., 2016), and (3) our main results of interest were conducted and replicated across two experiments.

### Experiment 1 fMRI Analysis

As stated in the section “General fMRI Methods” above, multiple regression analysis was performed on the time-series data at each surface node, for all nodes of the brain. There were separate event-related regressors for “sample,” “delay,” and “test” periods. Specific to Experiment 1, the “sample” regressor included the fixation, trial cue, left/right cue, and sample array (2.2 s total). The “delay” regressor only included the 8 s memory delay period, which was our primary event of interest. Finally, the “test” regressor included the test array/response and feedback display (1.8 s total).

### Experiment 2 fMRI Analysis

Specific to Experiment 2, the “sample” regressor included the fixation, trial type cue, and sample array (1.4 s total). The “delay” regressor only included the 8 s memory delay period, which was our primary event of interest. Finally, the “test” regressor included the test array/response and feedback display (1.6 s total).

## Results

### Experiment 1 Results

Of the entire sample of 32 participants, there was one participant in which we could not identify any early visual areas from the quadrant localizer data. We additionally excluded four participants whose MRI data contained significant spiking artifacts and one participant who had excessive head motion. Finally, participants whose behavioral accuracy on the WM task in the scanner was >2SD below the group mean were excluded (*N* = 1). Therefore, our final sample used in the analyses reported below was *N* = 25.

#### Behavioral Results

**FIGURE 3:**
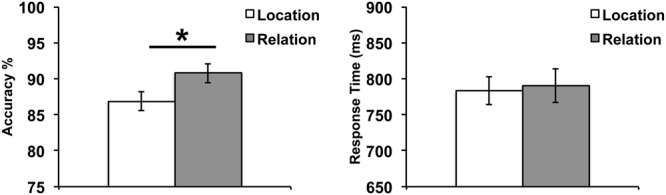
**Behavioral results illustrating accuracy **(left)** and RT **(right)**.** Error bars represent standard error of the mean. ^∗^*p* < 0.01.

To assess behavioral performance on our WM task, we tested paired-samples *t*-tests on accuracy and response time (RT) comparing Relation vs. Location trials (**Figure 3**). Results demonstrated no significant difference in RT between Relation and Location trials, *t*(24) = 0.46, *p* = 0.65. However, a significant difference in accuracy did emerge, *t*(24) = 3.02, *p* < 0.01, with accuracy being higher for Relation trials. While these accuracy results do suggest that Location trials were more difficult than Relation trials, the most important feature of our design is that in both trial types, participants were asked to encode and maintain only one piece of information (i.e., one location or one relation) and fMRI data was only analyzed for correct trials. Also, as can be seen in **Figure 3**, performance overall was quite high across both trial types.

#### Visual Cortex ROI Results

**FIGURE 4:**
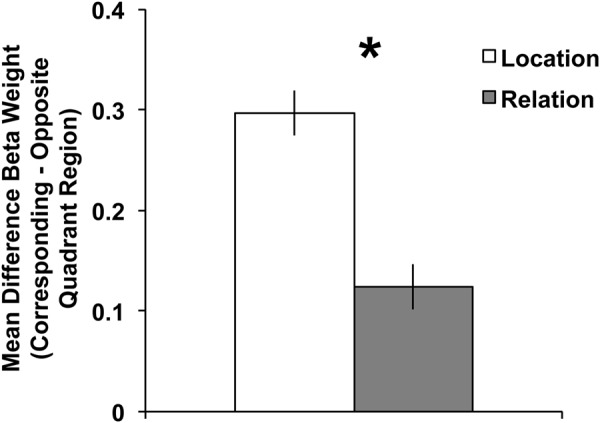
**Mean difference Beta weights (Corresponding Quadrant – Opposite Quadrant) from contralateral visual cortex ROIs (V1–V3) for Location and Relation trials.** The significant trial type × quadrant interaction suggests less quadrant-specific BOLD activity for Relation compared to Location trials. Error bars represent within-subject standard error of the mean. ^∗^*p* < 0.001.

As stated in the General Methods, our ROI analyses focused on the BOLD activity in the contralateral, “corresponding” quadrant (i.e., dorsal regions for a lower hemifield array or ventral regions for an upper hemifield array) relative to the contralateral, “opposite” quadrant (i.e., dorsal regions for an upper hemifield array or ventral regions for a lower hemifield array). Therefore, we tested a 2 (trial type: Location vs. Relation) × 2 (quadrant: corresponding vs. opposite) repeated-measures ANOVA on mean Beta weights. A main effect of trial type emerged, *F*(1,22) = 9.40, *p* < 0.01, with greater activity during Location compared to Relation trials. A main effect of quadrant also emerged, *F*(1,22) = 43.44, *p* < 0.001, with greater activity in the corresponding compared to the opposite quadrant, which demonstrates a quadrant-specific response overall to the maintained memoranda. Crucially, the trial type × quadrant interaction was significant, *F*(1,22) = 54.57, *p* < 0.001. **Figure 4** illustrates that the difference in the BOLD response between corresponding and opposite quadrants was greater for Location trials than Relation trials. Follow-up *t*-tests confirmed that BOLD activity in Location and Relation trials were significantly different in the corresponding quadrant, *t*(22) = 4.96, *p* < 0.001, but not for the opposite quadrant, *t*(22) = 1.05, *p* = 0.30.

#### Whole-Brain Results

**FIGURE 5:**
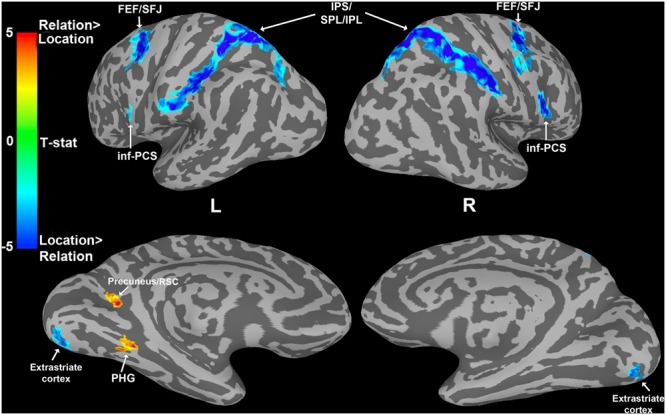
**Whole-brain analysis results showing a dissociation between Location and Relation trials during the WM delay period.** Cooler colors indicate areas significantly more active for Location than Relation trials. Warmer colors indicate areas significantly more active for Relation than Location trials. Abbreviations: frontal eye fields (FEF), superior frontal junction (SFJ), intraparietal sulcus (IPS), superior/inferior parietal lobule (S/IPL), inferior precentral sulcus (inf-PCS), retrosplenial cortex (RSC), and parahippocampal gyrus (PHG).

To find cortical areas that were more sensitive to maintaining either a spatial relation or a spatial location in WM, we contrasted Relation and Location trial delay period activity. As shown in **Figure 5**, (see **Table 1** for MNI coordinates) several bilateral areas demonstrated significantly greater BOLD activity for Location trials compared to Relation trials, including PPC regions [i.e., intraparietal sulcus (IPS), superior parietal lobule (SPL), and inferior parietal lobule (IPL)], frontal eye fields (FEF) and superior frontal junction (SFJ), inferior precentral sulcus (inf-PCS), and extrastriate cortex. These results are consistent with previous work demonstrating that SPL is involved in shifts of spatial attention (Yantis et al., 2002) and that IPS and FEF contain topographic maps of visual space (Hagler and Sereno, 2006; Silver and Kastner, 2009; Wang et al., 2014). Further, inf-PCS has been shown to contain topographic representation in a spatial WM task (Hagler and Sereno, 2006). The FEF and SFJ regions being activated more by Location trials here are consistent with areas previously shown to be specifically associated with WM for spatial locations in humans (Courtney et al., 1998).

**Table 1:** Experiment 1 MNI center of mass coordinates.

Region	*x*	*y*	*z*	Area mm^2^
Relation > Location				
Left precuneus/RSC	-29.8	-35.35	-26.65	118.61
Left PHG	-20.29	-42.52	-10.71	112.46
Location > Relation				
Left IPS/SPL	-31.98	-32.75	28.11	2664.84
Left FEF/SFJ	-23.5	9.24	34.18	840.28
Left extrastriate cortex	-20.68	-69.51	-31.7	334.18
Left inf-PCS	-54.79	21.83	12.47	101.17
Right IPS/SPL	33.67	-30.35	32.83	2656.74
Right FEF/SFJ	32.83	10.78	32.35	1101.24
Right extrastriate cortex	19.43	-67.77	-28.34	262.95
Right inf-PCS	52.38	23.92	3.11	618.96

Two regions demonstrated significantly greater delay period activation for Relation trials compared to Location trials: left parahippocampal gyrus (PHG) and left precuneus/retrosplenial cortex (RSC; **Figure 5**). Both the PHG and the precuneus/RSC have been shown to be involved in relational processing. For example, there is accumulating evidence that the MTL is involved in memory for relational information over short delays (Hannula et al., 2006; Olson et al., 2006; Piekema et al., 2006; Hartley et al., 2007). The precuneus has also been found to be involved in short-term memory processes related to relational information (Burgess et al., 2001; Wallentin et al., 2006) and the RSC has been shown to be involved in spatial navigation (for a review see, Vann et al., 2009). Distinguishing between the precuneus and RSC in humans may be difficult given their proximity, individual anatomical variation, and similar functional roles, therefore we refer to these two regions together throughout.

Given the significant difference in accuracy between the two trial types, one could argue that the areas more active for Location trials are sensitive to difficulty, which explains the dissociation in activity. A correlation analysis revealed that there was no significant relationship between accuracy and BOLD magnitude in any of the Location or Relation areas described above, all *R*s < 0.26, all *p*s > 0.07, uncorrected for multiple comparisons. This lack of a correlation between accuracy and BOLD activity in these Location and Relation regions suggests that the difference in accuracy level for the two trial types cannot explain the whole brain results. Further, we explicitly manipulated difficulty by using two WM loads in Experiment 2.

### Experiment 2 Results

Of the entire sample of 33 participants, one participant was excluded due to excessive motion during the scan. Also any participant whose behavioral accuracy for the WM task in the scanner was >2SD below the group mean was excluded (*N* = 1). Therefore, our final sample used in the analyses reported below was *N* = 31.

#### Behavioral Results

**FIGURE 6:**
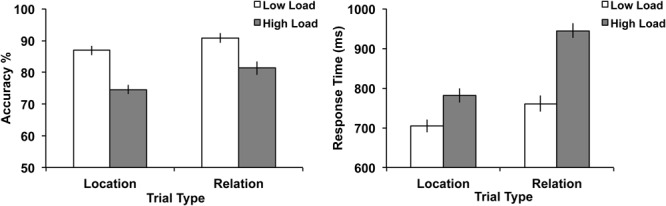
**Behavioral results illustrating accuracy **(left)** and RT **(right)** for both trial types and load.** Error bars represent standard error of the mean.

Accuracy and RT data were analyzed using a 2 (trial type: Location vs. Relation) × 2 (load: low vs. high) repeated-measures ANOVA. The results are shown in **Figure 6**. For accuracy, the main effect of load was significant, *F*(1,30) = 101.15, *p* < 0.001, with accuracy being higher on low load trials. The main effect of trial type was significant, *F*(1,30) = 22.25, *p* < 0.001, with accuracy being higher on Relation trials. The trial type x load interaction did not reach significance, *F*(1,30) = 2.55, *p* = 0.12. For RT, the main effect of load was significant, *F*(1,30) = 278.14, *p* < 0.001, with RT being faster on low load trials. The main effect of trial type was significant, *F*(1,30) = 64.87, *p* < 0.001, with RT being slower on Relation trials. The trial type x load interaction was also significant, *F*(1,30) = 51.90, *p* < 0.001, where the increased load slowed RT more in Relation trials than Location trials.

#### Visual Cortex ROI Results

As in Experiment 1, our ROI analyses focused on the BOLD activity in the contralateral, “corresponding” quadrant (i.e., dorsal regions for a lower hemifield array or ventral regions for an upper hemifield array) relative to the contralateral, “opposite” quadrant (i.e., dorsal regions for an upper hemifield array or ventral regions for a lower hemifield array). Therefore, we tested a 2 (load: low vs. high) × 2 (trial type: Location vs. Relation) × 2 (quadrant: corresponding vs. opposite) repeated-measures ANOVA on mean Beta weights. One participant’s data were excluded from ROI analyses due to mean Beta weights exceeding 2SD from the group mean. A main effect of quadrant emerged, *F*(1,29) = 43.48, *p* < 0.001, with greater activity in the corresponding compared to the opposite quadrant, which like Experiment 1 demonstrates a quadrant-specific response overall to the maintained memoranda. Neither the main effect of trial type nor of load reached significance, *p*s ≥ 0.28. The trial type x load interaction also did not reach significance, *p* = 0.14. As in Experiment 1, the trial type × quadrant interaction was significant, *F*(1,29) = 28.15, *p* < 0.001, with the difference in the BOLD response between corresponding and opposite quadrants being larger for Location trials than Relation trials. Further the load × quadrant interaction was also significant, *F*(1,29) = 66.55, *p* < 0.001, with a larger difference between corresponding and opposite quadrants for low load trials compared to high load. Finally, the 3-way trial type × load × quadrant interaction was significant, *F*(1,29) = 35.11, *p* < 0.001.

**FIGURE 7:**
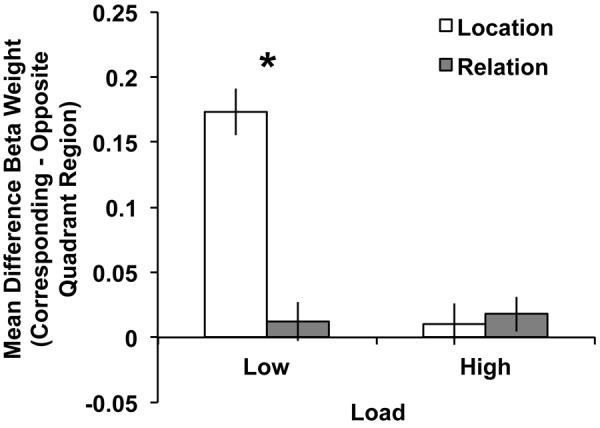
**Mean difference Beta weights (Corresponding Quadrant – Opposite Quadrant) from visual cortex ROIs (V1–V3) for Location and Relation trials under low (left) and high (right) load conditions.** For low load, the significant trial type × quadrant interaction suggests less quadrant-specific BOLD activity for Relation compared to Location trials, which replicates Experiment 1. Error bars represent within-subject standard error of the mean. ^∗^*p* < 0.001.

To explore this 3-way interaction we tested two separate 2 (trial type: Location vs. Relation) × 2(quadrant: corresponding vs. opposite) repeated-measures ANOVAs, one for each load. For low load, a main effect of quadrant emerged, *F*(1,29) = 78.33, *p* < 0.001, with more activity in the corresponding compared to opposite quadrant. The main effect of trial type did not reach significance, *F*(1,29) = 2.56, *p* = 0.12. Importantly, the trial type × quadrant interaction was significant, *F*(1,29) = 50.12, *p* < 0.001, with the difference in the BOLD response between corresponding and opposite quadrants being larger for Location trials than Relation trials. Follow-up *t*-tests confirmed that BOLD activity in low load Location and Relation trials were significantly different in the corresponding quadrant, *t*(29) = 3.96, *p* < 0.001, but not for the opposite quadrant, *t*(29) = 1.31, *p* = 0.20. As **Figure 7** illustrates, this low load result is a direct replication of Experiment 1’s ROI results. For high load, the main effect of quadrant approached significance, *F*(1,29) = 2.86, *p* = 0.10, with more activity in the corresponding compared to opposite quadrant. However, neither the main effect of trial type, *F*(1,29) = 0.07, *p* = 0.80, nor the trial type × quadrant interaction, *F*(1,29) = 0.16, *p* = 0.69, approached significance (**Figure 7**).

#### Whole-Brain Results

**FIGURE 8:**
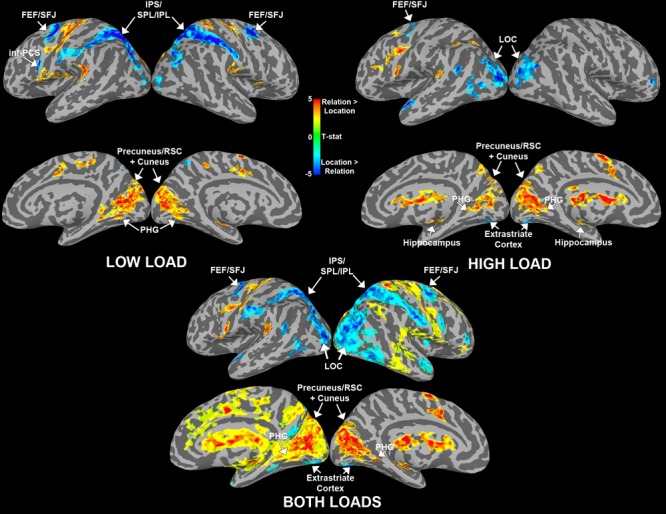
**Whole-brain results for the WM delay period shown by load.** Data are shown separately for low and high load (above) and averaged across load (below). Maps illustrate brain areas that were significantly more active for Relation (warmer colors) or Location (cooler colors) trials. Abbreviations: frontal eye fields (FEF), superior frontal junction (SFJ), intraparietal sulcus (IPS), superior/inferior parietal lobule (S/IPL), inferior precentral sulcus (inf-PCS), retrosplenial cortex (RSC), and parahippocampal gyrus (PHG).

For the whole-brain analysis we were interested in two main contrasts: (1) regions sensitive to maintaining spatial locations vs. relations and (2) regions sensitive to load for each trial type. First, to find cortical areas that were more sensitive to maintaining either a spatial relation(s) or a spatial location(s) in WM, we contrasted Relation vs. Location trial delay period activity for low and high load separately (**Figure 8**). For low load, we found that bilateral IPS/SPL/IPL, bilateral FEF/SFJ, and left inf-PCS were more active for Location trials compared to Relation trials. We also found that left precuneus/RSC and bilateral PHG and cuneus were more active for Relation trials compared to Location trials. These results replicate the findings in Experiment 1. We did find additional regions sensitive to Location and Relation trials that were not present in Experiment 1 (see **Figure 8**; **Table 2**). These regions may have emerged due to increased power in Experiment 2 due to a larger sample size and/or from the slight modifications made to the task to accommodate the load manipulation.

**Table 2:** Experiment 2 MNI center of mass coordinates.

Region	*x*	*y*	*z*	Area mm^2^
LOW LOAD				
Relation > Location				
Left peripheral visual cortex + cuneus + PHG	-20.71	-93.79	-11.62	1880.33
Left central sulcus	-60.07	-7.32	65.33	1163.7
Left inf frontal + insula	-81.96	19.10	-1.04	524.44
Left supramarginal gyrus	-87.97	-29.44	8.38	259.4
Left precentral gyrus	-78.79	19.06	38.54	120.06
Left Hippocampus	-41.60	-4.14	-46.93	126.67
Left anterior cingulate sulcus	-14.50	31.84	35.93	223.3
Left superior cingulate sulcus	-17.57	32.51	66.68	109.89
Left posterior cingulate sulcus	-15.99	16.65	43.13	98.9
Left precuneus/RSC	-18.41	-29.52	50.95	97.25
Right peripheral visual cortex + cuneus + PHG	25.64	-82.86	-7.15	2372.02
Right central sulcus	54.41	-7.99	70.37	299.21
Right supramarginal gyrus	84.84	-12.75	11.52	146.25
Right postcentral sulcus	47.39	-39.11	74.53	182.47
Right posterior cingulate sulcus	16.97	-12.62	56.10	278.61
Right anterior cingulate sulcus	15.03	29.21	41.68	261.16
Right insula	67.64	40.34	-14.32	97.37
Location > Relation				
Left IPS/SPL	-54.84	-60.81	43.97	2617.32
Left FEF/SFJ	-50.61	23.78	64.09	773.63
Left inf-PCS	-83.59	33.96	17.20	141.88
Left lateral occipital cortex	-54.69	-113.01	-13.83	131.58
Right IPS/SPL	56.25	-55.87	49.14	2848.68
Right FEF/SFJ	51.61	28.41	62.50	717.46
Right lateral occipital cortex	53.96	-113.05	-14.70	251.37
HIGH LOAD				
Relation > Location				
Right peripheral visual cortex + cuneus + PHG	24.46	-77.17	-14.20	825.58
Right thalamus and other subcortical regions	6.72	12.69	-10.32	963.14
Right parieto-occipital sulcus	26.53	-95.84	27.79	146.94
Right precuneus/RSC	20.42	-76.21	43.87	201.7
Right cuneus	19.72	-98.30	9.00	130.39
Right hippocampus	35.50	-5.26	-42.82	100.47
Left peripheral visual cortex + cuneus + PHG	-20.47	-89.96	-9.25	1645.75
Left thalamus and other subcortical regions	-9.08	3.35	-12.21	1046.46
Left cingulate sulcus	-16.55	39.15	55.39	672.71
Left supramarginal gyrus	-87.36	-30.04	5.66	117.18
Left superior IPS	-54.53	-73.52	45.39	141.26
Left precentral gyrus	-77.26	31.85	32.84	366.53
Left inferior central sulcus	-89.95	25.10	10.67	105.51
Left inferior frontal sulcus	-73.84	61.65	11.37	93.9
Left middle frontal gyrus	-50.71	42.62	59.09	127.15
Left precuneus/RSC	-19.31	-74.72	44.32	162.11
Left hippocampus	-39.57	-4.69	-46.33	124.58
Left inferior IPS	-66.62	-49.92	45.70	125.98
Location > Relation				
Right lateral occipital cortex	59.35	-97.55	8.19	1050.85
Right extrastriate cortex	42.99	-87.07	-42.14	173.27
Right inferior/anterior frontal sulcus	75.28	84.34	-6.66	101.83
Left lateral occipital cortex	-53.42	106.50	0.96	1272.33
Left anterior superior temporal sulcus	-89.56	16.21	-38.82	197.1
Left anterior occipital sulcus	-79.65	-73.79	-17.10	254.4
Left FEF/SFJ	-49.73	19.39	66.41	216.42
Left middle occipital gyrus	-73.98	-87.14	-13.01	112.6
Left superior temporal sulcus	-84.72	-56.76	11.33	107.7
Left extrastriate cortex	-44.36	-87.44	-44.24	142.58

For high load, we found that bilateral lateral occipital cortex (LOC), bilateral extrastriate cortex and left FEF/SFJ were more active for Location compared to Relation trials. Regions that were more active for Relation compared to Location included bilateral cuneus and precuneus/RSC, bilateral PHG and bilateral hippocampus. **Table 2** details all regions that were significantly activated for these particular contrasts. We also conducted a Relation vs. Location contrast averaged across both low and high loads to elucidate regions that differentiate these two trial types regardless of load. **Figure 8** shows that bilateral IPS/SPL/IPL, FEF/SFJ, and LOC were more active for Location trials; whereas bilateral precuneus/RSC, cuneus, and PHG were more active for Relation trials. These regions are consistent with both the separate load analyses and Experiment 1’s results. Of note, many of the regions listed in **Table 2** for the Relation vs. Location high load contrast were not seen in the low load contrast. Therefore, we were next interested in examining whether the same regions were sensitive to the load manipulation for the two trial types.

**FIGURE 9:**
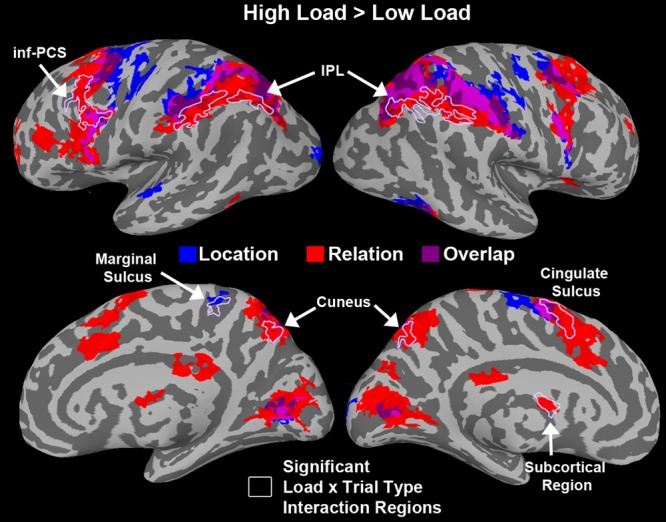
**Whole-brain results showing regions that were significantly more active for High vs. Low load during the WM delay period.** Blue regions are areas sensitive to load for Location trials only. Red regions are areas sensitive to load for Relation trials only. Purple regions are overlapping regions that were sensitive to load for both trial types. Regions that showed a significant load × trial type interaction are outlined in white. Abbreviations: inferior parietal lobule (IPL) and inferior precentral sulcus (inf-PCS).

We tested a load x trial type interaction using AFNIs 3dANOVA3 command to determine regions that were sensitive to load differentially for each trial type. **Figure 9** shows a conjunction map of regions sensitive to load for Relation trials, Location trials, and overlapping regions sensitive to load for both trial types. Regions that showed a significant load × trial type interaction are outlined in white in **Figure 9** and included bilateral IPL, left inf-PCS, bilateral cuneus, right marginal sulcus, left cingulate sulcus, and a left subcortical region. The bilateral IPL regions were sensitive to load only for Relation trials. The left inf-PCS region was also mostly sensitive to load for Relation trials (shown in red), but showed a small portion that was sensitive to load for both trial types (shown in purple). Finally, the bilateral cuneus regions were sensitive to load for Relation trials only, consistent with our trial type contrasts discussed above. While only these particular regions showed a significant load × trial type interaction, **Figure 9** shows all regions that were sensitive to load for Relation, Location, and both trial types.

## Discussion

Here we were interested in revealing the neural correlates underlying maintenance of spatial locations and spatial relations in WM. While previous work has suggested a distinction between these two types of memoranda, we sought to understand the role of early visual cortex in maintaining these two types of information and to investigate how load influences neural activity, as measured by fMRI, when locations and relations are held in WM. Across two experiments, we found that visual cortex was activated in a more quadrant-specific manner when one spatial location was being maintained as compared to when one spatial relation was being maintained in WM. Furthermore, we found a consistent set of brain regions that were more active when maintaining relation and location information under low load in both experiments. Under high load, we found some of the same regions as under low load that were sensitive to maintaining location or relation information, such as LOC and FEF/SFJ for location trials and precuneus/RSC and PHG for relation trials. However, we also found several regions that were sensitive to the relation vs. location contrast under high load that did not emerge under low load, such as left temporal regions for location trials and bilateral hippocampus and left frontal regions for relation trials. Finally, we found that there were distinct brain regions that were sensitive to load for location and relation trials, as well as overlapping regions that were sensitive to load for both types of information. Taken together, this evidence clearly suggests that the neural substrates underlying WM maintenance of spatial locations and relations are distinct from one another. However, the results also suggest that the neural representations of spatial information in WM change with load, which appears to change, somewhat, the nature of the dissociation between areas recruited for locations vs. relations.

Here we utilized a visuospatial WM task that required participants to maintain either spatial location or spatial relation information. Across both experiments we found that when one spatial location was maintained, early visual cortex demonstrated more quadrant-specific activation as compared to when one spatial relation was maintained. This finding is consistent with previous EEG work showing that there is increased posterior alpha power, which is thought to reflect suppression of sensory cortex, when a spatial relation is held in WM compared to a spatial location (Ikkai et al., 2014; Blacker et al., 2016). However, here our BOLD data illustrate that Relation activity (corresponding – opposite) was essentially at zero, which does not suggest suppression of visual cortex below baseline, but instead an elevation of relevant-quadrant activity for Location trials compared to Relation trials. Given the quadrant-specific stimulus presentation we used, our fMRI data suggest that WM for a spatial location activated the expected quadrant-specific activity in the contralateral corresponding visual cortex region compared to the opposite quadrant region. Our significant quadrant by trial type interaction indicates that this pattern was present to a greater degree for location compared to relation trials. Interestingly, in Experiment 2 when a high load condition was added, the visual cortex activation no longer showed this pattern of results. When multiple discrete items are stored in WM, it has been shown that the spatial configuration between items is also stored and influenced by both top-down cues like instructions and bottom-up grouping cues (Jiang et al., 2000; Gmeindl et al., 2011). It’s possible that increasing the load of our location trials resulted in an obligatory maintenance of the configural information between locations, which may have influenced the specificity of the activation in early visual cortex. In other words, it’s possible that by increasing load, our participants treated our location trials more like relation trials in the sense that they remembered the configural information. Indeed, this interpretation is consistent with our ROI results in that the activation patterns for high load location trials are similar to both low and high load relation trials. Future work could directly examine this possibility by manipulating both top-down and/or bottom-up grouping cues for our location trials.

While our main interest was in investigating the maintenance-related activity in early visual cortex, we also examined whole brain analyses in both experiments to illustrate regions sensitive to holding these two types of spatial information in WM and whether these same regions or others were also sensitive to load for relations and locations. Under low load in both experiments we found that several bilateral regions were more active for location compared to relation trials, including IPS/SPL/IPL, FEF/SFJ, and inf-PCS. These regions have been shown to be retinotopically organized and involved in spatial WM (Courtney et al., 1998; Hagler and Sereno, 2006; Silver and Kastner, 2009; Wang et al., 2014). Thus these findings nicely complement our results in early visual cortex that spatial locations were maintained in a more sensory-based and spatially specific manner. Furthermore, in both experiments cuneus and/or precuneus/RSC regions emerged as being more active for relation compared to location trials, as well as MTL regions including the PHG in both experiments and the right hippocampus in Experiment 2. There is accumulating evidence that the MTL (Hannula et al., 2006; Olson et al., 2006; Piekema et al., 2006; Hartley et al., 2007) and the precuneus/RSC (Casey et al., 1998; Burgess et al., 2001; Wallentin et al., 2006; Vann et al., 2009) are involved in memory for relational information over short delays. However, this previous work has only used sensory-based types of relations such as the relational binding of object identity with its location. Our findings suggest that these MTL and precuneus/RSC and cuneus regions are involved in WM for a broad range of relational information including more abstract information that is not tied to the original sensory percept.

Under high load we saw some of the same regions as under low load including more activity in FEF/SFJ for locations compared to relations and more activity in MTL areas for relation compared to location. However, by increasing the load, the way the information was maintained in WM may have changed thus eliciting different regions sensitive to relation and location trials. Indeed, previous work has shown that increasing the WM load and the degree to which items can be grouped or chunked can alter the underlying neural activity (Bor et al., 2003). In addition to demonstrating that distinct regions are supporting the maintenance of spatial relations and locations in WM, we investigated regions sensitive to load for both of these information types. **Figure 9** shows that several regions including, IPS, FEF/SFJ, and inf-PCS, were sensitive to load and tended to show a gradient of areas sensitive to relation load, areas sensitive to location load and areas sensitive to load for both. However, only a subset of these regions in **Figure 9** showed a significant load × trial type interaction. Only bilateral IPL, left inf-PCS, bilateral cuneus, and other regions not found in previous analyses showed a significant sensitivity to load that differed between the two trial types. Taken together, this evidence again suggests that these two forms of spatial WM rely on distinct subregions of these areas that are known to be critical for WM and sensitive to load.

Another way to consider our findings here may be in the context of the existing literature on egocentric vs. allocentric spatial processing. One might consider our spatial location trials to be egocentric in that participants are maintaining locations in relation to themselves and where they fall on the screen presented to them. Likewise, our relation trials may be more allocentric insofar as the locations must be maintained in relation to each other (i.e., the relation between the two or three circles). Indeed it has been shown that allocentric short-term memory is impaired in patients with bilateral hippocampal lesions, but egocentric short-term memory is spared (Holdstock et al., 2000). This is consistent with our results here that show that areas in the MTL are more active for maintaining spatial relations compared to locations. Moreover in a navigation task Jordan et al. (2004) found that participants who used a more allocentric strategy activated parahippocampal and hippocampal regions more and those who used an egocentric strategy activated IPS to a greater degree. Thus our results are consistent with the neuropsychological and neuroimaging literature that demonstrates distinct neural underpinnings for egocentric vs. allocentric spatial processing. Our novel results add to this work by demonstrating that this distinction also exists for spatial WM. Our results also suggest that, if indeed our location vs. relation distinction is analogous to the egocentric vs. allocentric distinction others have proposed, then the distinction is more general than just a shift in coordinate system. There appear to be multiple types of spatial information that can be maintained in WM and they can be selectively or jointly recruited depending on the task demands and memory load.

By having low load conditions in both Experiments 1 and 2 we were able to confirm the replicability of the results of Experiment 1. All of the regions identified in Experiment 1 showed similar activation patterns in Experiment 2. We did, however, see additional regions sensitive to our relation vs. location contrast in Experiment 2. While the task did change slightly between experiments (i.e., colored circles instead of gray scale, and a unilateral memory array instead of bilateral), we attribute these additional findings to our larger sample size in Experiment 2 and thus the additional power afforded by the sample size. Further, one unexpected result emerged in Experiment 2 that was not present in Experiment 1 and is worth noting further. In both low and high load, there were significant bilateral clusters in what looks like early visual cortex showing greater activity for relation compared to location trials. At first glance, this appears at odds with our ROI results, but the clusters specifically fall outside the bounds of the regions sensitive to the quadrants within which we presented the memory array. In other words, the clusters correspond to visual cortex that represents peripheral visual space beyond where we displayed our stimuli. These clusters are also quite large and seem to encompass peripheral visual cortex, cuneus and PHG regions. These latter two regions are unsurprising given Experiment 1 and the existing literature, but peripheral visual cortex was unexpected. While previous work has shown that central attentional load can modulate activity in peripheral visual cortex (Schwartz et al., 2005), to the best of our knowledge this has not been shown or explored previously in the context of WM and may represent an interesting future direction.

Together, our two experiments here suggest that abstract and sensory memoranda can be maintained in WM distinctly and may compete with one another for neural resources. While there may be instances where sensory and non-sensory information act in a cooperative or hierarchical relationship, here we designed our task to put these two types of information in competition with one another, which resulted in distinct activations of neural systems underlying WM. For example, while everyday activities like spatial reasoning and/or navigation may require WM for both of spatial locations and spatial relations, our evidence here suggests that these types of spatial information can be held in WM distinctly. The process by which these two forms of spatial information are combined, as in the example of navigation, will be an important future direction. Finally, these distinct systems and their competitive interactions may have important implications regarding individual variability in higher cognitive function.

## Author Contributions

KB was involved in all aspects of the project, including its conception, study design, data acquisition, data analysis, data visualization, data interpretation, and manuscript preparation. SC was involved in project conception, study design, data interpretation, and manuscript preparation.

## Conflict of Interest Statement

The authors declare that the research was conducted in the absence of any commercial or financial relationships that could be construed as a potential conflict of interest.

## Footnotes

1 The size of the quadrants used in Experiment 2 was slightly more narrow horizontally (0.75°) and set slightly closer to the meridians (0.2° vs. 0.4° in Experiment 1). The quadrant localizer stimuli were adjusted accordingly to match the same space.
